# Identification of Essential Sequences for Cellular Localization in BRMS1 Metastasis Suppressor

**DOI:** 10.1371/journal.pone.0006433

**Published:** 2009-07-30

**Authors:** José Rivera, Diego Megías, Carolina Navas, Jerónimo Bravo

**Affiliations:** 1 Signal Transduction Group, Centro Nacional de Investigaciones Oncológicas, Madrid, Spain; 2 Confocal Microscopy Unit, Centro Nacional de Investigaciones Oncológicas, Madrid, Spain; Dresden University of Technology, Germany

## Abstract

**Background:**

Breast cancer metastasis suppressor 1 (BRMS1) reduces the number and the size of secondary tumours in a mouse model without affecting the growth of the primary foci upon its re-expression. Knockdown of BRMS1 expression associates with metastasis. The molecular details on BRMS1 mechanism of action include its ability to function as a transcriptional co-repressor and consistently BRMS1 has been described as a predominantly nuclear protein. Since cellular distribution could represent a potential mechanism of regulation, we wanted to characterize BRMS1 sequence motifs that might regulate its cellular distribution. According to its amino acids sequence, BRMS1 contain two putative nuclear localization signals, however none of them has been proved to work so far.

**Methodology/Principal Findings:**

By using well known in vivo assays to detect both nuclear import and export signal, we have characterized, in the present study, one functional nuclear localisation signal as necessary and sufficient to promote nuclear transport. Additionally, the outcome of a directed yeast two-hybrid assay identify importin α6 as a specific partner of BRMS1 thus speculating that BRMS1 nuclear import could be specifically mediated by the reported nuclear transporter. Besides, the combination of a computational searching approach along the utilization of a nuclear export assay, identified a functional motif within the BRMS1 sequence responsible for its nuclear export, that resulted not affected by the highly specific CRM1 inhibitor Leptomycin-B. Interspecies heterokaryon assay demonstrate the capability of BRMS1 to shuttle between the nuclear and cytosolic compartments

**Conclusions/Significance:**

Our results show for the first time that BRMS1 contains both nuclear import and export signals enabling its nucleo-cytoplasmic shuttling. These findings contributes new data for the understanding of the BRMS1 functions and allow us to speculate that this phenomenon could represent a novel mechanism for regulating the activity of BRMS1 or its associated cytosolic partners

## Introduction

Breast cancer metastasis suppressor 1 (BRMS1), one of the members of a recently described family of proteins known as metastasis suppressors, specifically inhibit the development of secondary foci without affecting the growth of the primary tumour. These results were demonstrated by ectopic expression into highly metastatic cells in an experimental in vivo assay (reviewed in [1]).

Underlying mechanisms of action proposed for BRMS1 include facilitation of cell-cell communication [2], interaction with HDAC complex components [3], [4], [5], shutting-down of PI3K signalling [6] and gene expression inhibition by targeting NFκB [7]. Recently, more data have provided insight into potential new mechanism by which BRMS1 could inhibit metastasis progression [8].

Part of the wide range of molecular mechanisms involving BRMS1 might be caused by its role in transcriptional repression [5], [7], [8], [9], [10]. Nuclear localisation is therefore important to perform BRMS1 cellular functions and possibly inhibit metastasis progression.

Analysis of the BRMS1 amino acid sequence identified two coiled-coil motives at residues 51–81 and 147–180, and two putative canonical monopartite nuclear localization signals (NLSs) located at amino-acid residues 198–205 (NLS1) and 239–245 (NLS2). However, their functionalities have not been experimentally confirmed yet, nor have been the possibility of BRMS1 diffusion to the nucleus.

Even though its expression has been largely restricted to the nucleus, previous work [8] and unpublished data in our laboratory have led us to raise the hypothesis of a plausible presence of BRMS1 in the cytosol. Evidence comes from protein-protein interaction found using FRET analyses, as well as by the identification of cytosolic partners by yeast two-hybrid assays (submitted). The latter results have also been shown by other authors [11] reinforcing our conjecture. The presence of a putative nuclear export signal (NES) capable of complementing the alleged nuclear localisation and therefore enabling BRMS1 to shuttle between both compartments has not been postulated. If this hypothesis is correct it could imply that the activity of BRMS1 might be modulated by compartmentalisation. It would also shed light into a new more complicated mechanism of action for its anti-metastatic activity. Therefore, this issue represents an important molecular question for the understanding of cancer genetics and metastasis involving BRMS1.

The active transport of proteins through the nuclear pores is commonly mediated by nuclear import and export receptors belonging to the karyopherin family [12]. The transport receptors bind to specific signals known as NLS and NES. Cargo proteins exposing a NES are usually recognized and bound by CRM1, mediating the export towards the cytoplasm of cellular and viral proteins as well as ribonucleoproteins [12], [13]. CRM1-mediated export is inhibited by Leptomycine-B (LMB) [14]. Typical NES are rich in large hydrophobic amino acids with characteristic spacing among them [15]. So far, 67 high-confidence NES have been reported, ΦX_2_ΦXΦ (where Φ = L, I, V, F, M and X is any amino-acid) being the most conserved pattern [16] which deviates from the previously accepted consensus ΦX_2–3_ΦX_2–3_ΦXΦ [15], [17].

Protein transport towards the nucleus is determined by the presence of a single (monopartite) or two short stretches of basic amino acids spaced by 10–12 residues (bipartite). Monopartite sequences are exemplified by the SV40-NLS one (PKKKRKV).

Import receptors recognize and bind cargoes in the cytosol followed by its delivering inside the nucleus. One such import receptor is importin α6, a nuclear transporter for NLS-containing proteins. This protein features an N-terminal domain that is responsible for importin β binding and necessary for nuclear translocation through the nuclear pore, eight armadillo repeats involved in the NLS recognition and binding, and a C-terminal acidic region whose function remains unclear.

Here we report that the NLS1 motif of the BRMS1 protein, but not NLS2, is necessary and sufficient for nuclear transport. Furthermore, yeast two-hybrid results suggest that nuclear import of BRMS1 might be mediated by importin α6. Besides, we demonstrate that BRMS1 has the capability to migrate between nucleus and cytoplasm and define precisely the motifs responsible for this shuttling. A functional nuclear export sequence (NES) was identified between residues 74–91 of BRMS1. The nuclear-cytoplasm shuttling of BRMS1 represents a previously non-reported, possible mechanism for regulating the activity of BRMS1 or its associated cytosolic partners. The identified NES is CRM1-independent, since the CRM1-inhibitor LMB did not block the exit of exogenously expressed protein.

## Results

### The region of BRMS1 encompassing residues 198 to 246 is able to transport a heterologous protein into the nucleus

We want to characterize the properties of putative nuclear localization signals (NLSs) present within the BRMS1 protein sequence. Although it seems possible that a protein without its own NLS enters the nucleus via co-transport with a partner harbouring one, many nuclear proteins encode their own NLSs.

Analysis and prediction of cellular localization signals based on BRMS1 amino-acid sequence by the PSORT II server (http://psort.nibb.ac.jp/form2.html) [18] reveals the presence of two putative NLSs at residues 198–205 (PPSKRKKA) and 238–245 (PQKRKSD) fulfilling the loose consensus of the classical monopartite NLS which requires a lysine in the P1 position followed by basic residues in positions P2 and P4 to yield a consensus sequence of K K/R X K/R [19].

**Figure 1:**
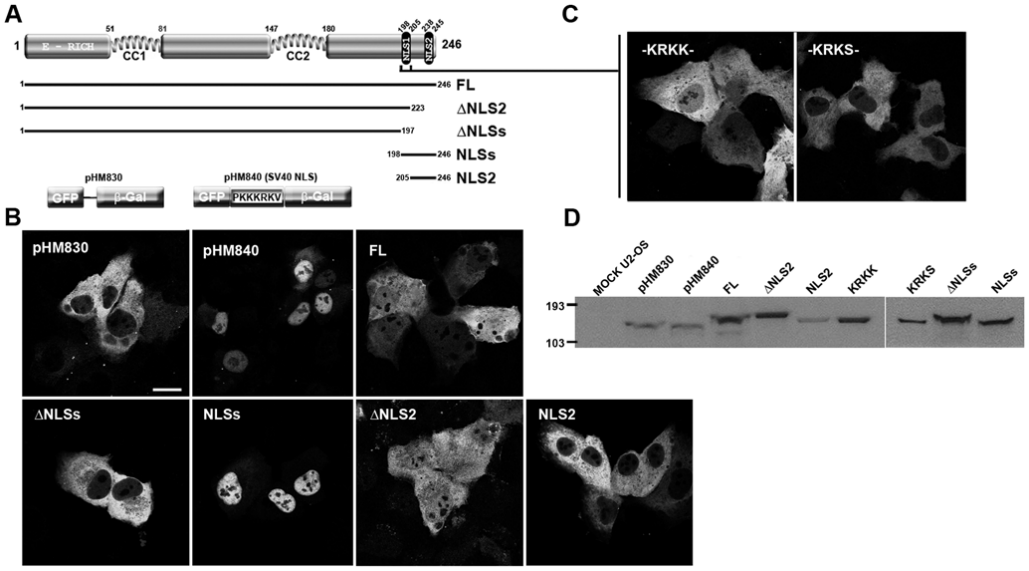
NLS1 is necessary and sufficient for nuclear targeting of BRMS1. (A) Schemes of full length (FL) BRMS1, deletion mutants and their controls (pHM830 and pHM840). Plasmids express a triple GFP/insert/β-Gal fusion protein. (B) Confocal images of U-2 OS transfected cells expressing GFP-β-Gal NLS constructs. (C) A construct encompassing the residues KRKK from NLS1, resemble the location pattern of the FL. KRKS, represents a point mutant where the last positively charged residue shifted to uncharged. (D) Total cell lysates from U-2 OS untransfected (mock) or transfected cells with the indicated constructs were analysed by Western Blotting using an anti-GFP antibody. Molecular markers are shown in kDa.

To check whether NLS1 and NLS2 are required for BRMS1 nuclear import, we engineered truncated forms of BRMS1 (Figure 1A) and inserted them into the NLS mapping vector pHM830 [20]. The resulting fusion protein is large enough to prevent its passive diffusion to the nucleus, since its size is far above the diffusion limit for the mammalian nuclear pore complex [21]. This system has extensively been used for the unambiguous identification of the NLS activity of both conventional [20] and non conventional import signals [22].

**Figure 2:**
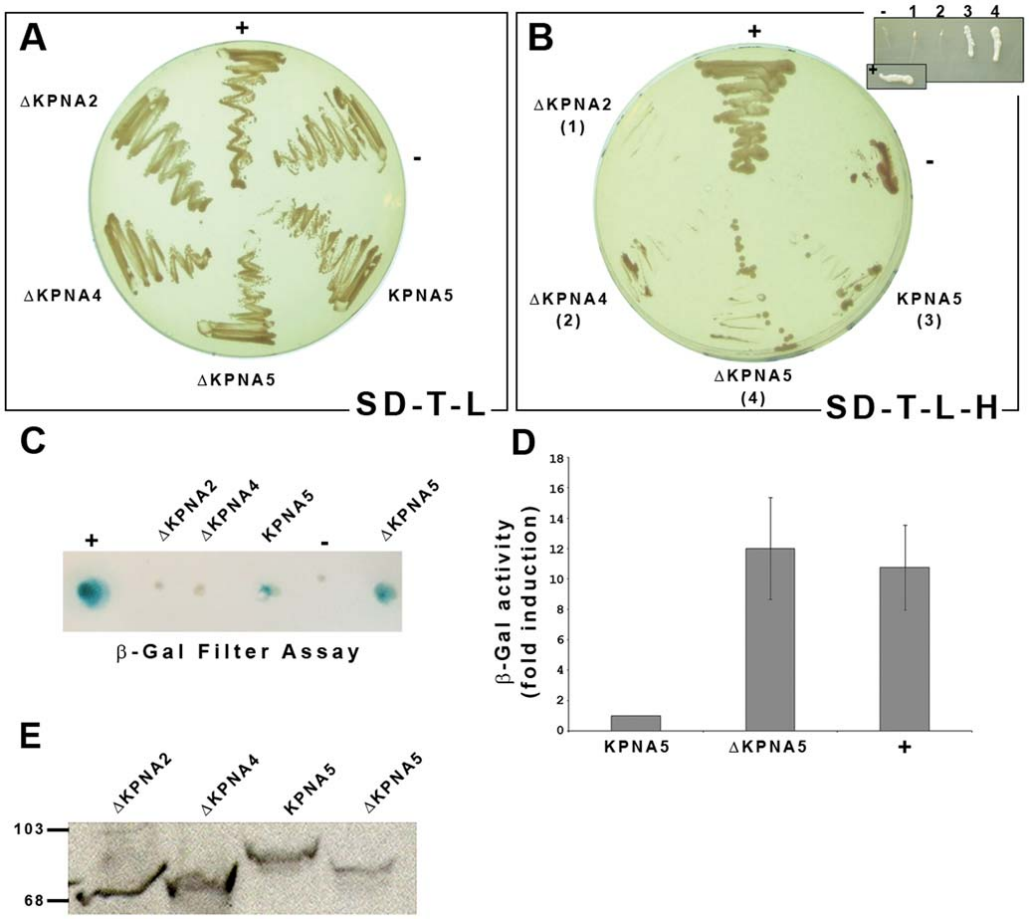
BRMS1 specifically interacts with importin α6 in an *in vivo* assay. Yeast cells co-transformed with a vector encoding the entire BRMS1 and a combination of plasmids encoding the complete sequence of importin α6 (KPNA5) or deletion mutants lacking the auto-inhibitory domains (ΔKPNA5); importin α1 (ΔKPNA2); or importin α3 (ΔKPNA4), were grown on selective medium (SD-L-T) to assess co-transformation (A) or plated on highly stringent media (B). Inset shows a clearer picture for growth capacity of different co-transformant yeast indicated in brackets. Positive control (+) as previously reported [24]. Negative sign (−) indicates co-transformation with an empty vector. Colonies grown on SD-L-T-H, were assayed for β-Gal activity by a colony lift filter (C) or quantitative liquid assay (D) where activity is represented as a fold induction compare to the basal activity shown in yeast expressing KPNA5. Error bars represent S.E.M of three independent experiments. (E) Western blot analysis of the different HA tagged karyopherins probed with anti-HA antibody. Molecular markers are in kDa.

Constructs were transiently transfected into U2-OS cells and GFP signal expression was analysed 24 h later. A plasmid containing the entire BRMS1 sequence (pHM830/BRMS1 FL) showed a diffuse staining both in the nucleus and the cytosol (Figure 1B and Figure S1). Over-expression of a truncated version encompassing amino-acid 2–197, thus lacking both nuclear signals (pHM830/ΔNLSs), showed a diffuse distribution of fluorescence restricted to the cytosol (Figure 1B and Figure S2). The latter staining pattern was also observed after over-expression of pHM830 empty vector, which is unable to import the GFP towards the nucleus (Figure 1B and FigureS2). Interestingly, a construct containing residues 197–246 (pHM830/NLSs) encompassing the two clusters of basic amino-acid was able to relocate the fusion protein into the nucleus as efficiently as a positive control (pHM840), containing the monopartite NLS of the SV40 (Figure 1B and Figure S2).

These data demonstrate that the C-terminal end of BRMS1 (residues 197–246) indeed contains a functional NLS, which is necessary and sufficient to target a heterologous protein towards the nucleus.

### NLS1 is necessary and sufficient to mediate nuclear import in an in vivo assay

To further characterize the NLSs and shed light into their relative contributions to the nuclear import of the FL BRMS1 protein, we engineered two more constructs containing either NLS1 (pHM830/ΔNLS2, encompassing residues 2–223) or NLS2 (pHM830/NLS2, harbouring residues 205 to the C-terminus) and analyzed their cellular locations after over-expression. The fusion protein expressed from the pHM830/NLS2 plasmid did not migrate to the nuclear compartment, showing a sole cytosolic location pattern. Conversely, the protein expressed from the pHM830/ΔNLS2 construct displayed the characteristic pattern revealed by the FL construct (Figure 1B and Figure S2). Taken together these results suggest that NLS1, but not NLS2, is capable of mediating the nuclear import of a heterologous protein, underscoring its role as a nuclear import signal.

To precisely determine the amino acid responsible for nuclear import, oligonucleotides encoding the KRKK sequence found within the NLS1 region, were annealed and cloned into the pHM830 vector (pHM830/KRKK). As shown in Figure 1B and Figure S2, upon transient transfection of this plasmid into mammalian cells, the expression pattern resembles that shown by the construct containing the full NLS1 but also the one displayed by the vector encoding the entire BRMS1. Interestingly, a construct with the fourth lysine residue of the NLS consensus sequence replaced by an uncharged serine (-KRKS-) as found in the putative NLS2, showed an expression pattern indistinguishable from the one observed during over-expression of the pHM830 empty vector, abolishing completely the nuclear location. Altogether, these results demonstrate that NLS2 from BRMS1 does not play a role as a signal for targeting protein(s) into the nucleus, whilst the NLS1 is necessary and sufficient for functioning as a classical monopartite NLS.

All the over-expression experiments for NLS characterization were also carried out in HeLa (Figure S3), and MDA-MB-231 cells (data not shown), resulting in subcellular location patterns indistinguishable for those observed in U2-OS transfected cells, thus suggesting that the NLS signal present in BRMS1 operates via a general mechanism that is cell-type independent. Furthermore, the expression of all these fusion proteins was verified by western blot confirming that they were correctly synthesized, present in a stable form and their relative molecular mass were in agreement with the expected molecular weights values (Figure 1D).

### Importin α6 but not α1 nor α3 interacts with BRMS1 in an *in vivo* yeast two-hybrid assay

Different importins, also named karyopherins (KPNAs), are known to bind several cargoes in the cytoplasm and transport them into the nucleus. To identify the mechanism of BRMS1 nuclear transport, we analyzed by a yeast two-hybrid assay the interaction of BRMS1 protein with several importins, belonging to different subfamilies based on the similarity of their primary structure [23].

To achieve this goal, the AH109 *S. cerevisiae* strain was co-transformed with a yeast vector containing the BRMS1 FL sequence (pAS2.1/BRMS1) and one of the yeast expression plasmids encoding the entire importin α6 (pACT2/KPNA5), importin α1 (pACT2/KPNA2) or α3 (pACT2/KPNA4). Colonies were grown on selective agar medium lacking tryptophan and leucine (SD-T-L) to confirm the presence of both expression plasmids (Figure 2A). Positive colonies were re-streaked onto high selective plates lacking histidine as a first reporter gene (SD-T-L-H). After 4–5 days of incubation co-transformed cells over-expressing importin α6 (KPNA5) were capable to grow (Figure 2B). However, yeast colonies over-expressing importin α1 (KPNA2) or importin α3 (KPNA4) failed (data not shown). A colony-lift filter β-galactosidase assay was performed to confirm that only those yeast cells over-expressing importin α6 and BRMS1 showed β-Gal expression (Figure 2C). These results suggest that full-length BRMS1 specifically interacts with KPNA5, while it does not with the other two tested importin α transporters.

To determine whether BRMS1 protein is capable of auto-activation, yeast cells were co-transformed with the bait protein along with an AD empty vector, grown on selective medium (SD-T-L-H) and further assayed for β-Gal expression. As shown in Figure 2C no β-Gal expression was detected verifying that Gal4 BD/BRMS1 itself does not activate transcription of any of the reporter genes in yeast. Furthermore, AH109 strain was co-transformed with two previously characterized interacting proteins [24] and their interaction was used as a positive control (Figure 2A-D).

Since the N-termini of importin α proteins have been shown to constitute auto-inhibitory domain [25], [26], we generated Gal4 AD importin α fusion proteins lacking their respective auto-inhibitory domains and co-transformed them into yeast cells together Gal4 BD BRMS1 construct.

As shown in Figure 2B, yeast over-expressing mutated versions of importin α1 or α3 failed to grow in the absence of histidine in the growth media and thus were unable to express β-galactosidase in a colony lift filter assay (Figure 2C). Interestingly, co-transformed yeast over-expressing an N-terminal mutant of importin α6 grew in SD-T-L-H plates at a higher extent that the FL version (Figure 2B). In order to quantitatively measure the β-galactosidase activity of these two positive constructs we used ONPG as a substrate in a liquid culture colorimetric assay that confirmed the stronger interaction between BRMS1 and N-terminal-deleted importin α6 (Figure 2D). The stability and proper folding of all the tested constructs expressed was confirmed by western blot, using a specific monoclonal antibody against the HA tag (Figure 2E).

Altogether, these results support the hypothesis that KPNA5 (importin α6) and not KPNA2 (importin α1) nor KPNA4 (importin α3) specifically might mediate the nuclear transport of BRMS1 towards the nucleus.

### BRMS1 displays nucleo-cytoplasmic shuttling in heterokaryons

Preliminary results in our lab [8] showed that even though the majority of the over-expressed BRMS1 protein was present within the nucleus we repeatedly detected a weak but unambiguous presence of BRMS1 in the cytosol, suggesting that at least transiently it might play a functional role in that compartment. Given the fact that we identified a functional NLS within BRMS1, as shown in Figure 1, and that some interaction with cytosolic proteins has been observed (Rivera et al. submitted) and reported [11], we hypothesized that a fraction of the protein could be exported from the nucleus to the cytosol.

**Figure 3:**
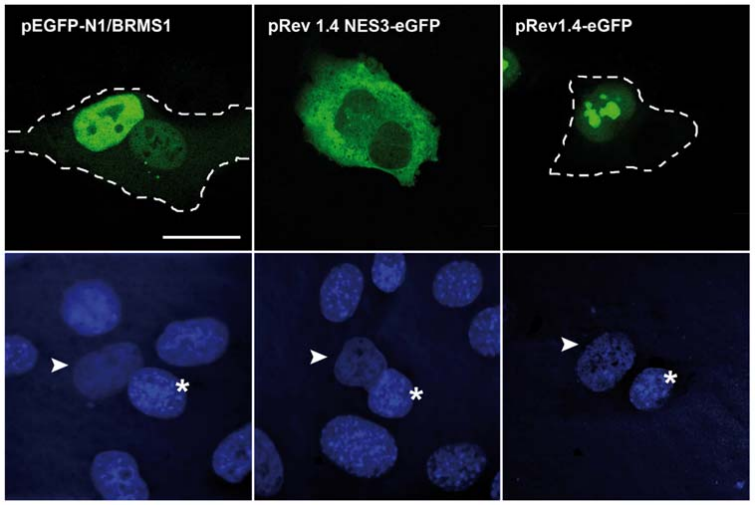
BRMS1 protein migrates between nuclei in interspecies heterokaryon. Human HeLa cells were transfected with a plasmid encoding the entire BRMS1 sequence (pEGFP-N1/BRMS1), a non-shuttling (pRev1.4-eGFP) or a competent shuttling vector (pRev1.4NES3-eGFP). Then, HeLa cells were co-cultured with mouse myoblast. After fusion, cells were fixed and stained with Hoescht. Cycloheximide was added to the cell culture medium before fusion and maintained throughout culture of heterokaryons. Cellular distribution of GFP fusion proteins (top) and nuclear staining (bottom) were analyzed. Mouse and human nuclei are marked by asterisk and arrowhead respectively. Heterokaryons are marked with a dashed line. Bar represents 20 µm.

To test this hypothesis we performed an interspecies heterokaryon assay, extensively used to assay nuclear protein export [27], [28]. Human HeLa cells transfected with an expression vector encoding the full-length BRMS1 fused to GFP (pGFP-N1/BRMS1 FL), were co-cultured with mouse C2C12 cells until adequately spread. Since cycloheximide was added to the cell culture media, the fluorescent signal was limited to GFP-fusion proteins synthesized prior to cell fusion. Interspecies heterokaryons were easily distinguishable by their nuclear staining pattern upon cells analysis by confocal microscope, where human and mouse nuclei appeared diffusely stained or spotted respectively (Figure 3).

Thirty minutes after heterokaryon formation, the BRMS1-GFP fusion protein was detected not only in the human but also in the murine nuclei of fused cells (Figure 3) indicating that BRMS1 can be exported out of the human nuclei and imported into the mouse one. In a parallel assay, shuttling of a competent export construct containing the powerful NES from HIV-1 Rev protein (pRev1.4NES3-eGFP) was observed (Figure 3). Conversely a non-competent export construct expressing GFP fused to a Rev mutant [29] remained in the human nuclei (Figure 3), thus confirming that shuttling of BRMS1 is not due to a diffusion process.

This result suggests that BRMS1 is actively exported from the nucleus and can translocates from nucleus to cytoplasm although so far, the endogenous protein has been mainly observed in the nuclei. We speculate that a prolonged cytosolic presence of BRMS1 protein may not be necessary, though a transient cytosolic location is likely necessary for accomplishing its physiological function.

### Identification of a putative nuclear export sequence in BRMS1

Once the capability to migrate from the nucleus to the cytosol was assessed, we wondered whether BRMS1 would harbour a nuclear export sequence (NES).

**Figure 4:**
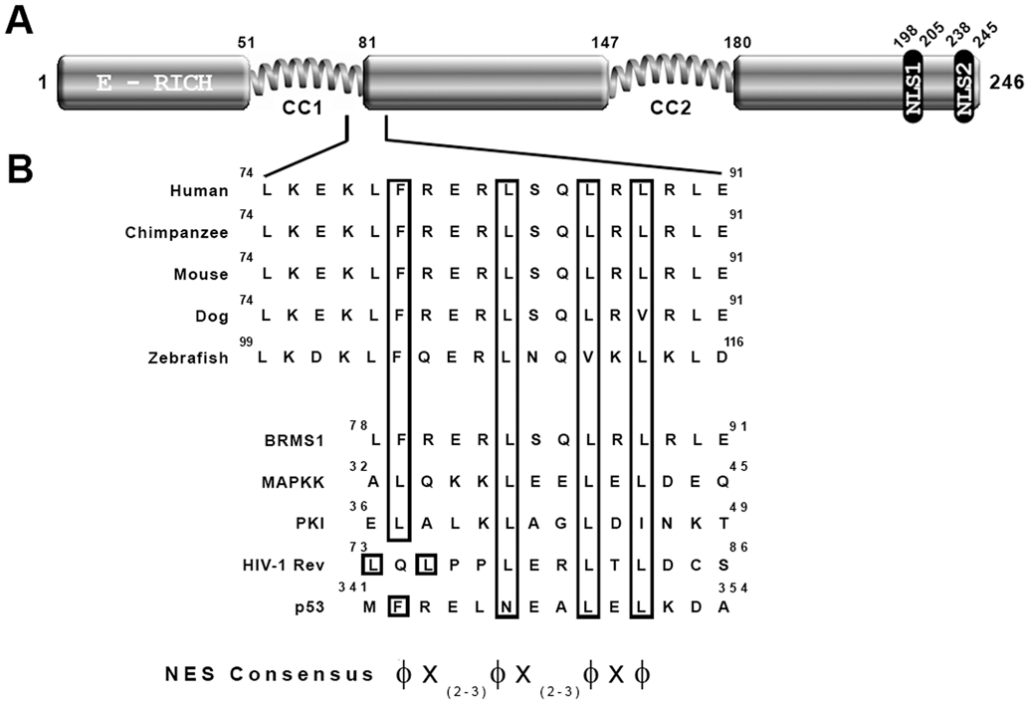
BRMS1 comprise a putative NES motif that compare to leucine-rich NES. (A) Schematic representation of the BRMS1 structure showing the putative NES sequence boundaries identified by NetNES 1.1 server. (B) Alignment of conserved hydrophobic residues (in boxes) from different BRMS1 orthologs. Numbers refer to amino acid residues. Human putative NES-BRMS1 sequence is compared with known NES, ranging from highest (MAPKK; PKI and Rev) to the weakest (p53) activity. A generally accepted loose consensus sequence, where X represents any amino acid and Φ any large hydrophobic residue (L, I, V, F, M) is shown.

We inspected its entire amino-acid sequence looking for a leucine, or any other large hydrophobic rich region similar to those previously identified as the transport signals that can mediate nuclear export [30], [31]. We noticed a large cluster of hydrophobic residues closely spaced in the amino terminal half of the protein that would fulfil the loose consensus of a leucine-rich NES. This result was confirmed after submission of BRMS1 sequence to the NetNES 1.1 database server [16]. The server assigned NES score values over the threshold for residues L^83^ to L^88^, which in addition to an over-representation of D, E and S residues suggest that this region conforms to the established criteria for a NES [30]. As shown in Figure 4 the alignment of all the BRMS1 orthologs deposited in the databases, identified by blastp search [32] demonstrates the high conservation of this candidate NES sequence among the different species, including the regularly conserved spacing between the critical residues for export function.

### Analysis of the candidate NES sequence by an in vivo assay

**Figure 5:**
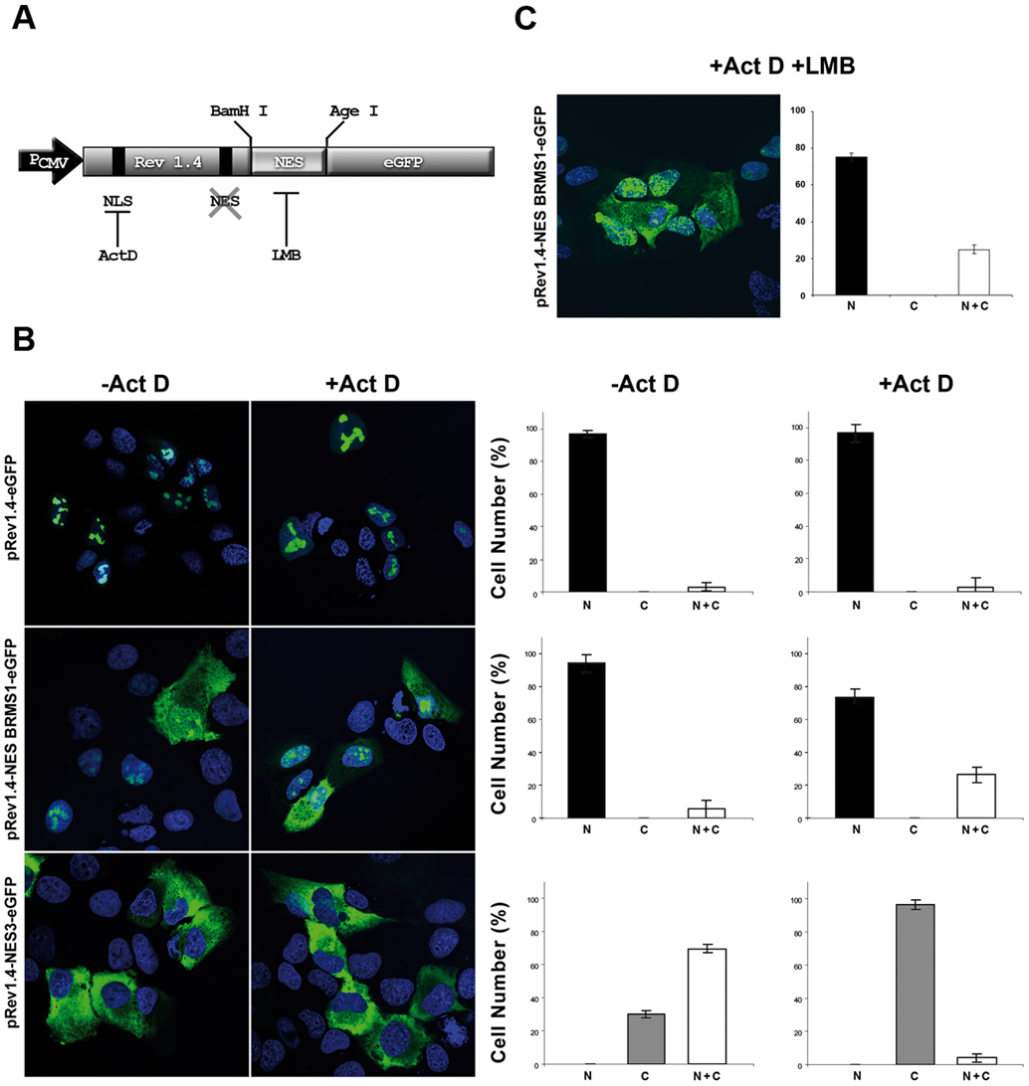
BRMS1 contains a functional nuclear export motif independent of CRM1. (A) Scheme of the plasmid system used for testing the putative BRMS1 NES motif. Disrupted endogenous NES of Rev protein (Rev1.4) allows the *in vivo* analysis capability of a *BamHI/AgeI* inserted sequence fused to eGFP. P_CMV_: CMV promoter. Representative confocal images of U-2 OS transfected cells with indicated plasmids untreated (−) or treated (+) with ActD (B) or Act-D + LMB (C). Fixed cells were scored for nuclear (N), citosolic (C) or diffuse (N+C) GFP location. Graphics show mean values of percentage of positive cells with S.E.M across three independent experiments.

To test the export activity of the identified candidate NES in BRMS1, we generated a construct encoding 18 residues (^74^LKEKLFRERLSQLRLRLE^91^, hydrophobic residues are underlined) inserted between the export deficient Rev1.4 sequence and eGFP (Figure 5A), rendering the pRev1.4-BRMS1-eGFP plasmid. That insert size has been found to be optimal for export activity [29]. Constructs were transiently over-expressed into different cell types. The use of Act-D has been reported to prevent nucleolar accumulation and nuclear import of Rev protein thereby provoking a cytosolic accumulation of NES-containing protein [33]. Therefore, we treated transfected cells with Act-D to facilitate the detection of weak NES signals [29].

Hence, the activity of NES-BRMS1 was evaluated by the ability of the NES containing fusion protein to shift from the nucleus towards the cytosol in U-2 OS (Figure 5B) or HeLa (Figure S3) transfected cells. A cell-counting approach was applied by scoring cells according to the distribution of the GFP fusion protein as exclusively nuclear (N), cytosolic (C), or diffuse (N+C) before and after Act-D treatment. At least 150 cells per sample (431 for pRev1.4/NES-BRMS1) were analyzed in this way across three independent experiments. As expected, the pRev1.4-eGFP negative control was found exclusively in the nucleus (∼97%), whereas the positive control pRev-NES3-eGFP was detected almost exclusively (97%) in the cytoplasm upon Act-D treatment (Figure 5B).

In basal conditions, without Act-D treatment, U-2 OS cells over-expressing NES-BRMS1-eGFP protein displayed a diffuse N+C staining slightly higher (5.6%) than that found in cells transfected with pRev1.4-eGFP negative control vector (3.2%), and clearly less cytoplasmic presence than observed for the positive control construct (pRev-NES3-eGFP) (Figure 5B). However, unbiased scoring of more than 400 cells revealed that the percentage of cells with diffuse N+C staining increased up to 26% following Act-D treatment (Figure 5B), while the negative control remained at 3% under these conditions.

To determine the relative strength of the BRMS1-NES an arbitrary scale ranging from 1+ to 9+ (the strongest) was applied [29]. Our results classify NES-BRMS1 as a very weak signal (+1) according to this NES scoring system.

Same approach was used with different cell lines (U-2 OS, HeLa and MDA-MB-231) but only shown in U-2 OS cells (Figure 5B). Interestingly, the candidate NES from BRMS1 maintained a similar degree of activity in all cell lines tested (data not shown) indicating that this effect is not cell type specific.

### Export of BRMS1 is not CRM1-dependent

CRM1 is an export receptor involved in nucleo-cytoplasmic exchange of several shuttling proteins [34] capable of binding cargo molecules containing a canonical NES [15]. To investigate whether CRM1 is involved in the nuclear export of NES-BRMS1, human U-2 OS cells transiently expressing the pRev1.4NES BRMS1-eGFP construct, were treated with the highly specific CRM1 inhibitor Leptomycin B (LMB). Thirty min later, Act-D was added to the culture medium to facilitate the detection of recombinant protein in the cytosol.

A positive control is able to relocate the fusion protein from the nucleus, even without Act-D treatment, towards the cytoplasm upon Act-D treatment. Such translocation is abolished when LMB is added to the culture media (Figure S4). Interestingly, we found that LMB treatment of cells had no significant effect on nuclear to cytoplasm shuttling of the reporter protein after transfection of pRev1.4/NES BRMS1-GFP plasmid (Figure 5C) independently of the cell type (data not shown) and of the presence or not of Act-D (Figure 5B). This observation suggests that nuclear export of BRMS1 is mediated through a CRM1-independent pathway.

## Discussion

In this study, we determined the capability of BRMS1 to mediate nucleo-cytoplasmic shuttling and identified the responsible signals.

Nuclear localization signals within BRMS1 have been characterized by an *in vivo* assay. Simultaneous disruption of NLS1+NLS2 abolishes nuclear location of a heterologous protein. Besides, a construct encompassing both NLS showed an exclusive nuclear pattern concluding that residues 197–246 are essential for nuclear location. In addition, this construct displays a stronger fluorescence within the nucleus compared to BRMS1-FL, suggesting that conformation of the properly folded (as shown in Figure 1D) FL fusion protein might somehow impair its nuclear transport, or that BRMS1 contains another signal(s) able to shuttle the protein back to the cytosol. The former observation becomes especially true after comparison of the pattern showed by a BRMS1 protein merely GFP attached and the very large BRMS1 protein expressed in the pHM830 system (Figure S1). An NLS1-deletion construct was exclusively expressed in the cytoplasm. Moreover, a pHM830/ΔNLS2 construct results in the same pattern shown by the FL-construct, suggesting that NLS2 is dispensable for nuclear allocation. Altogether we conclude that NLS1 constitute a functional, necessary and sufficient nuclear signal. A construct over-expression only four residues (^201^KRKK), within NLS1 motif shifts the fusion protein towards the nucleus as efficiently as the entire BRMS1 protein. Besides, a substitution of the fourth residue for an uncharged serine rendered the construct not competent for nuclear import. Precisely, the KRKS peptide is present within the NLS2 motif, underscoring its inability for nuclear import.

Our results largely prove the functionality of BRMS1-NLS1 and the inability of BRMS1-NLS2 as import signals by themselves. However, it is worth to consider that upon protein folding both motifs could be brought close enough to allow them to work cooperatively as a stronger motif. This possibility is endorsed by the observation of the strongest nuclear localization for a construct including both motives. It should be noted that our results do not exclude that BRMS1 could enter the nucleus as part of a complex with other protein(s).

We also shed light on the mechanism of BRMS1 nuclear translocation. Analysis of human importins responsible for such transport concludes that only KPNA5 showed significant interaction with BRMS1 upon a yeast two-hybrid assay. Since KPNA5 has been reported to interact with ANP32A, an acidic phosphoprotein involved in proliferation, differentiation and apoptosis [35] and with p27Kip1, a cyclin-dependent kinase inhibitor involved in G1-phase arrest [36] we hypothesized that the KPNA5/BRMS1 complex might also regulate other cellular functions apart from nuclear import of BRMS1.

Previous reported observations on our lab [8] as well as transiently BRMS1 over-expressing cells along the present work, show a consistent and repetitive presence of certain amounts of BRMS1 in the cytosol. Given that a mechanism for nuclear import exists, we hypothesized the possibility of an export signal in the BRMS1 sequence. Computer searching, identified a domain (residues 74–91) that fulfilled the proposed consensus NES motif [16]. Score values for important L-residue were above for L^30^ in BRCA1 [37] and L^149^ from FGF-1 [38], crucial residues for export activities. The BRMS1-NES motif is highly conserved, conforming not only to NES consensus motif but also to well-known NES sequences. Its functional role was assessed *in vivo*. Transfection of human cells with a NES-BRMS1-GFP fusion construct, showed a slight difference when compared with a non-competent construct in protein distribution of non-treated cells. Only 5.6% of transfected cells partially shift fluorescent protein towards the cytosol while the negative construct show similar values (3%). However, after inhibition of the nuclear re-import of NLS-Rev fusion proteins with Act-D, more than 26% of BRMS1-NES transfected cells showed a partial shift of GFP-protein towards the cytosol, whereas the non-competent construct maintained same levels as in non-treated cells. Similar effect was reported for well-known NES-containing proteins [29]. Scoring of transfected cells categorize BRMS1-NES as a functional, very low activity signal, as efficient as p53 and HDM2, but less active than proteins which completely shift the GFP-fusion protein in the absence of Act-D.

CRM1 binds cargoes containing a leucine-rich motif, with low affinity as compared with other exportin receptors [15] ensuring an efficient release. In order to understand if BRMS1-NES fusion protein is dependent on CRM1 export, transfected cells were treated with CRM1 inhibitor, and GFP distribution scored. Cells transfected with a NES-competent construct completely blocked the exit of GFP-fusion protein after LMB treatment (Figure S4). Unexpectedly, subcellular location was unaltered upon treatment of pRev1.4-BRMS1-NES-eGFP transfected cells. This effect was maintained when the protein re-import was also blocked by Act-D in co-treated (LMB+Act-D) cells (Figure 5C). Thus, we conclude that BRMS1 contains a functional CRM1-independent NES.

Eukaryotic cells separate transcription/replication from translation by compartmentalization into nucleus and cytosol respectively. The majority of proteins with nuclear function are actively transported based on its binding to specific karyopherin receptors usually by the presence of NLS and NES signals. In the absence of a specific retention mechanism based on the high affinity of a protein for nuclear components, all nuclear proteins shuttle, considering export as a default pathway [28]. In such a case the distribution of a protein will not be directed, at least not exclusively, by the import/export competence of the protein itself but also by its capability to bind to nuclear partners. In this sense, BRMS1 strongly interact with the Sin3-HDAC complex [3], [5] recruiting BRMS1 to specific DNA regions. This interaction provides a plausible explanation along with the weakness of the BRMS1-NES for the predominant nuclear location of BRMS1. The fact that BRMS1 has access to the cytoplasm, even transiently, might be important for integrating nuclear functions with those occurring in the cytoplasm, constituting a major event in the regulation of both activities.

It is worth to mention that apart from being significant for a better characterization of BRMS1 protein itself, insights into the mechanism and regulation of its nucleo-cytoplasmic shuttling capability might be pivotal from the point of view of a potential clinical/therapeutic approach. Many of reported shuttling proteins are involved in signalling and cell growth [39], [40] and their incorrect subcellular location related to the development of different types of cancers [41], [42]. As transcription occurs in the nucleus, the activity of different transcription factors can be regulated by their subcellular distribution. Thus, identification of molecules that could affect BRMS1 protein location might prove effective in controlling cell growth.

In conclusion, our findings suggest that BRMS1 shuttles between nucleus and cytoplasm mediated by a novel, weak but functional NES region. Moreover, we provide strong evidence that NLS1 is necessary and sufficient for BRMS1 nuclear transport. Finally, the identification of KPNA5 as BRMS1 partner has also been demonstrated. Since BRMS1 molecular functions are highly dependent on nuclear localisation, our results possibly suggest another level of regulation for BRMS1 biological mechanisms. Future studies should evaluate to what extent the reported nucleo-cytoplasmic shuttling might affect BRMS1 dependent anti metastatic properties. We speculate that BRMS1 activity by import/export transport could serve as a potential regulatory mechanism playing a biological role which functional/clinical implications are not yet understood.

## Materials and Methods

### Cell culture

U-2 OS, HeLa, MDA-MB-23 human and C2C12 mouse myoblast were from American Type Culture Collection (ATCC). Cells were grown in Dulbecco's modified Eagle's medium (Gibco) supplemented with 10% foetal bovine serum (EuroClone) in the presence of penicillin, streptomycin and 2 mM L-glutamine (Gibco) as recommended. Transfections were performed using Lipofectamine2000 (Invitrogen). After 24 h, cells were untreated or treated with Leptomycin B (LMB) (10 ng mL^−1^) or ActinomycinD (Act-D) (5 µg mL^−1^) for 3 h. Cycloheximide (15 µg mL^−1^) was added 30 min prior additional treatments to ensure that fusion protein is not due to *de novo* synthesis. Former products were from Sigma.

### Heterokaryon assay

HeLa cells grown on cover-slips were transfected with NES-expression vectors. After 24 h C2C12 mouse cells were co-cultured for 3 h, cycloheximide was added (100 µg mL^−1^) for 30 min. Heterokaryons were formed with 50% (w/v) PEG solution (Sigma-Aldrich; Inc) for 2 min at RT. Subsequently, the cells were extensively washed, and further incubated at 37°C in presence of cycloheximide. The cells were fixed 30 min later with 4% PFA, stained with Hoechst33258 (50 µg mL^−1^, Sigma-Aldrich; Inc) and mounted in Mowiol 4–88 (Calbiochem).

### Nuclear localization assay

The assay was based on the pHM830 eGFP–β-Galactosidase triple fusion plasmid [20]. Amplicons obtained by PCR using specific primers were ligated into pHM830 vector. BRMS1 cDNA full length clone (IRALp962L0425Q2) was obtained from the I.M.A.G.E. Consortium [43]. Five different constructs were engineered encompassing: the entire gene, residues 198–246 containing both putative NLSs (pHM830/NLSs), lacking the 48 (pHM830/ΔNLSs) or the last 23 C-terminal end residues (pHM830/ΔNLS2), and residues from 205 to 246 (pHM830/NLS2). Numbering is based on human sequence (BC_009834). More than 200 transfected cells were observed across three independent experiments.

### Nuclear export assay

Nuclear to cytoplasm *in vivo* assays were carried out as described [29]. Briefly, mammalian cells were transfected with an export-defective pRev(1.4)-eGFP protein, a competent nuclear export construct (pRev1.4-NES3-eGFP) or an engineered construct encompassing residues 74–91 from BRMS1 (pRev1.4-NES BRMS1). Relative export activity was measured 24 h later by scoring nuclear (N), cytosolic (C) or diffuse (N+C) location of fusion proteins (>200 cells) ranging from 1+ (weakest) to 9+ (strongest) activity.[29]. Candidate NES insert (residues 74 to 91), was prepared by annealing specific oligo-deoxy-nucleotides and cloned as *BamHI/AgeI* (pRev1.4-NES BRMS1-GFP). Cells were fixed, mounted and observed 24 h post-transfection.

### Fluorescence microscopy

Cells were visualized with a Leica TCS-SP5 confocal laser microscope equipped with HCX-PL-APO 63×lbd.BL (1.4NA) oil-immersion objectives and UV/argon lasers for DAPI/GFP detection. Scoring of fusion proteins distribution was as mentioned above. Independent assays were analyzed with Metamorph software (Universal Imaging Corp).

### Yeast two-hybrid assay

Human BRMS1 entire gene was PCR amplified, cleaved (*EcoRI/PstI*), inserted into Gal4-DNA binding domain (BD) of pAS2.1 (Clontech) vector (pAS2.1/BRMS1) and used as a bait to test interaction with human α importins fused to the Gal4 activation domain (AD) (pACT2/importin α6; pACT2/importin α1 and pACT2/importin α3). Deletion mutants for auto-inhibitory domains (lacking 60 N-terminal residues) were also generated. PCR-templates were from CNIO collection. Constructs were co-transformed by lithium acetate into AH109 yeast strain [44]. Transformant-colonies grown on high stringency selection media lacking, tryptophan, leucine and histidine (SD-T-L-H) were subjected to a colony-lift filter assay. Briefly, colonies transferred to a filter, were disrupted and β-Gal activity detected with Z-buffer containing 0.27% β-mercaptoethanol and 5-bromo-4-chloro-3-indolyl-β-D-galactopyranoside. Quantitative activity determination was performed using o-nitrophenyl-β-D-galactopyranoside (ONPG). None of the interactions tested were prone to self-activation. Chemicals and protocols were from Sigma-Aldrich and Yeast Handbook Clontech respectively.

## Supporting Information

Figure S1 Confocal images of GFP distribution after over-expression in U-2 OS cells of BRMS1 protein merely fused to the N-terminus of GFP (A) or as fusion protein in the pHM830 triple fusion plasmid (B).
(4.25 MB TIF)

Figure S2 A) Fluorescence intensity profile along a line crossing the cell body of U-2 OS transfected cells with the indicated GFP-β-Gal NLS constructs. Profiles show intensity of eGFP expression (green line) and nuclear staining (blue line). Values are normalized to 1. B) Merged image of ΔNLSs over-expressing cells where the intensity profile along a representative area of cell body (dashed line) is shown as an example. Profiles were recorded using the LAS AF 1.8.2v acquisition software of a Leica TCS-SP5 confocal microscope
(5.93 MB TIF)

Figure S3 Confocal images of transfected HeLa cells expressing GFP-β-Gal NLS constructs and their controls. Distribution of GFP fusion proteins is shown. KRKK, represents a construct encompassing precise residues from NLS1 of BRMS1 protein resembling the location pattern of the full length (FL). KRKS is a point mutant where the last positively charged residue shifted to uncharged, which was unable to relocate the heterologous protein to the cell nucleus.
(6.37 MB TIF)

Figure S4 The pRev1.4-eGFP and pRev1.4-NES3-eGFP fusion proteins were transiently over-expressed in U-2 OS cells. Cell samples were untreated (-) or treated (+) with LMB to assess CRM1 transporter inhibition.
(6.13 MB TIF)
