# Supplementary figures and images for: Identification of Essential Sequences for Cellular Localization in BRMS1 Metastasis Suppressor
Source: PLoS One. 2009 Jul 30;4(7):e6433. doi: 10.1371/journal.pone.0006433 (PMC2713406; doi:10.1371/journal.pone.0006433)

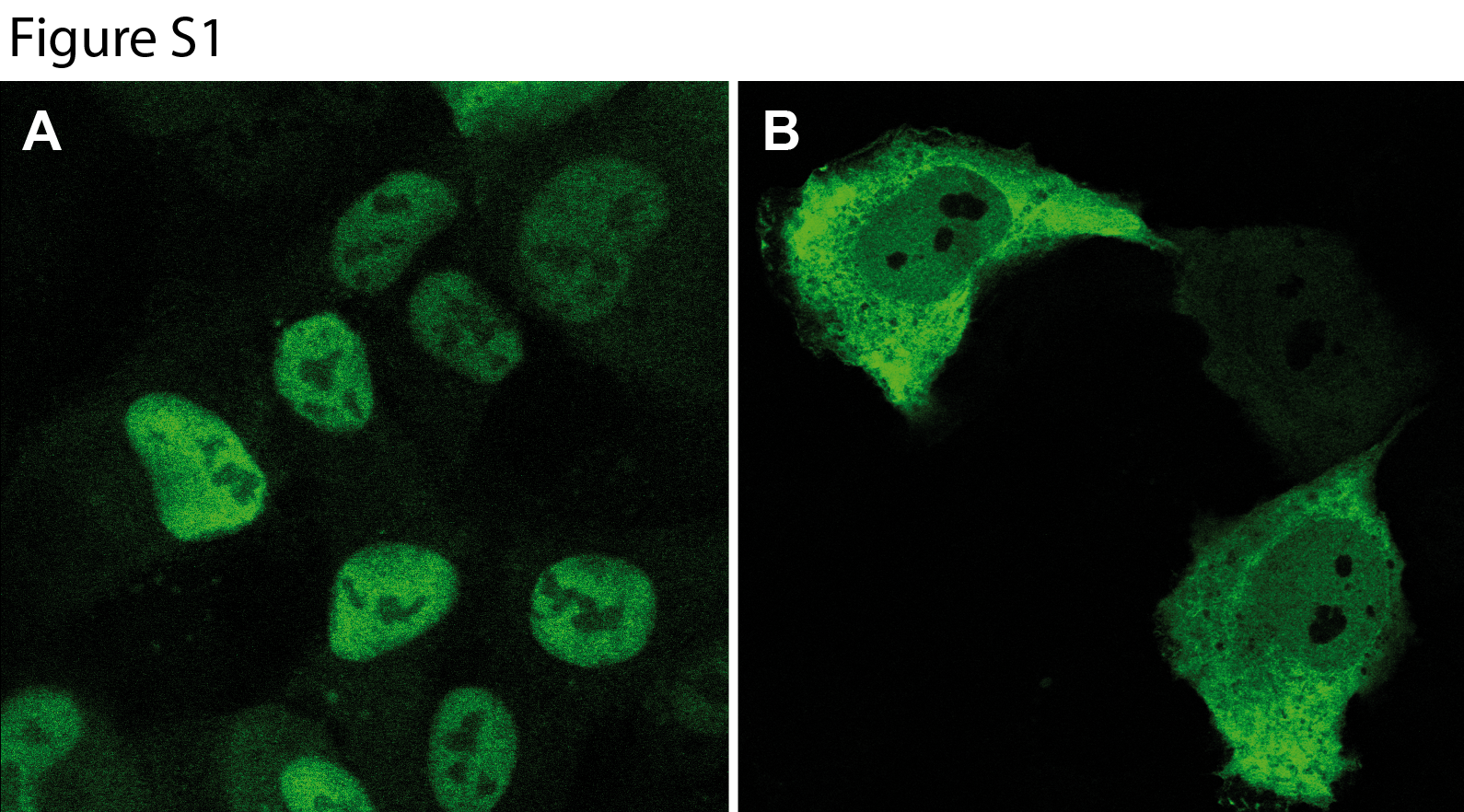

Supplement: Figure S1 — Confocal images of GFP distribution after over-expression in U-2 OS cells of BRMS1 protein merely fused to the N-terminus of GFP (A) or as fusion protein in the pHM830 triple fusion plasmid (B). (4.25 MB TIF) [file pone.0006433.s001.tif]

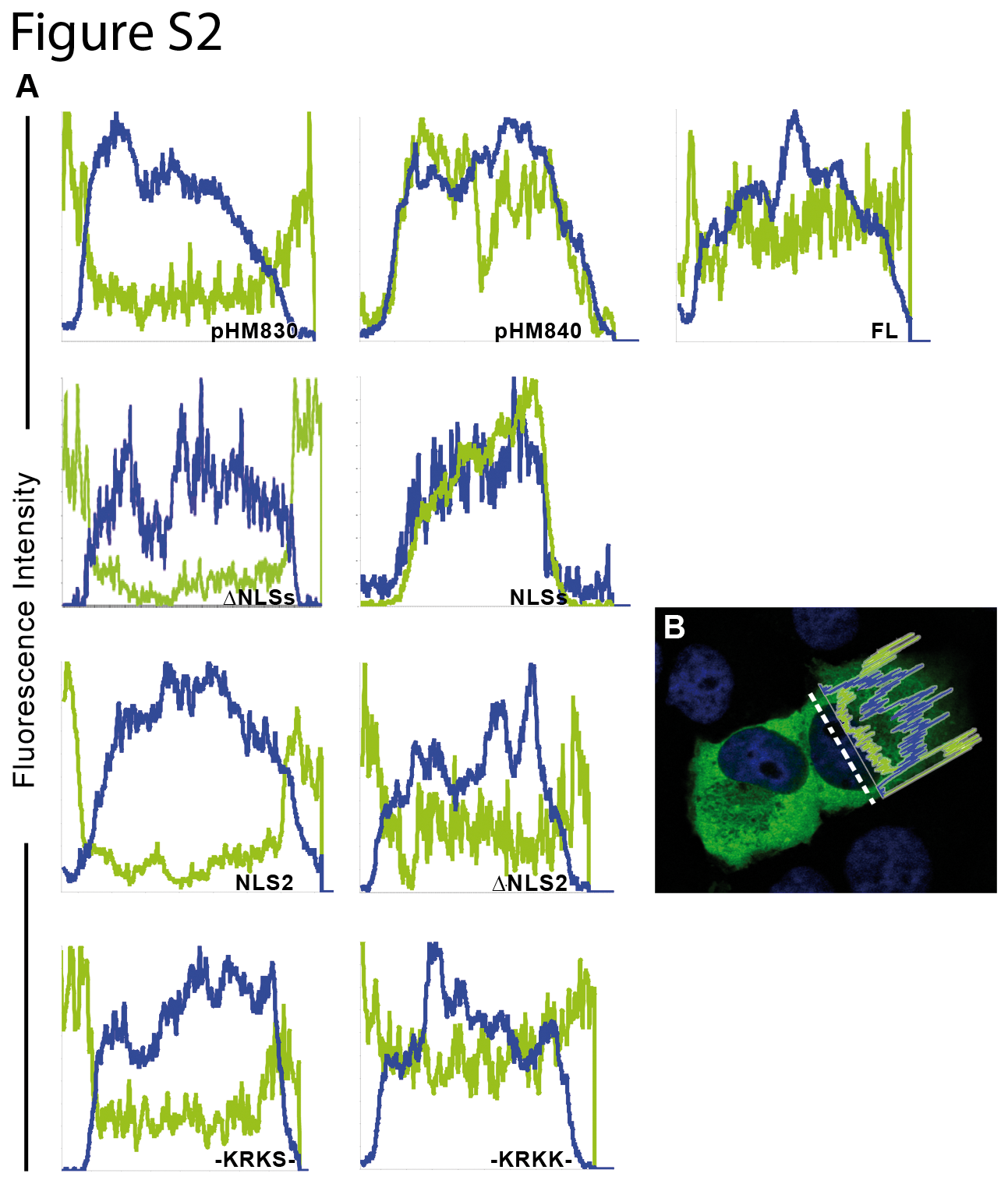

Supplement: Figure S2 — A) Fluorescence intensity profile along a line crossing the cell body of U-2 OS transfected cells with the indicated GFP-β-Gal NLS constructs. Profiles show intensity of eGFP expression (green line) and nuclear staining (blue line). Values are normalized to 1. B) Merged image of ΔNLSs over-expressing cells where the intensity profile along a representative area of cell body (dashed line) is shown as an example. Profiles were recorded using the LAS AF 1.8.2v acquisition software of a Leica TCS-SP5 confocal microscope (5.93 MB TIF) [file pone.0006433.s002.tif]

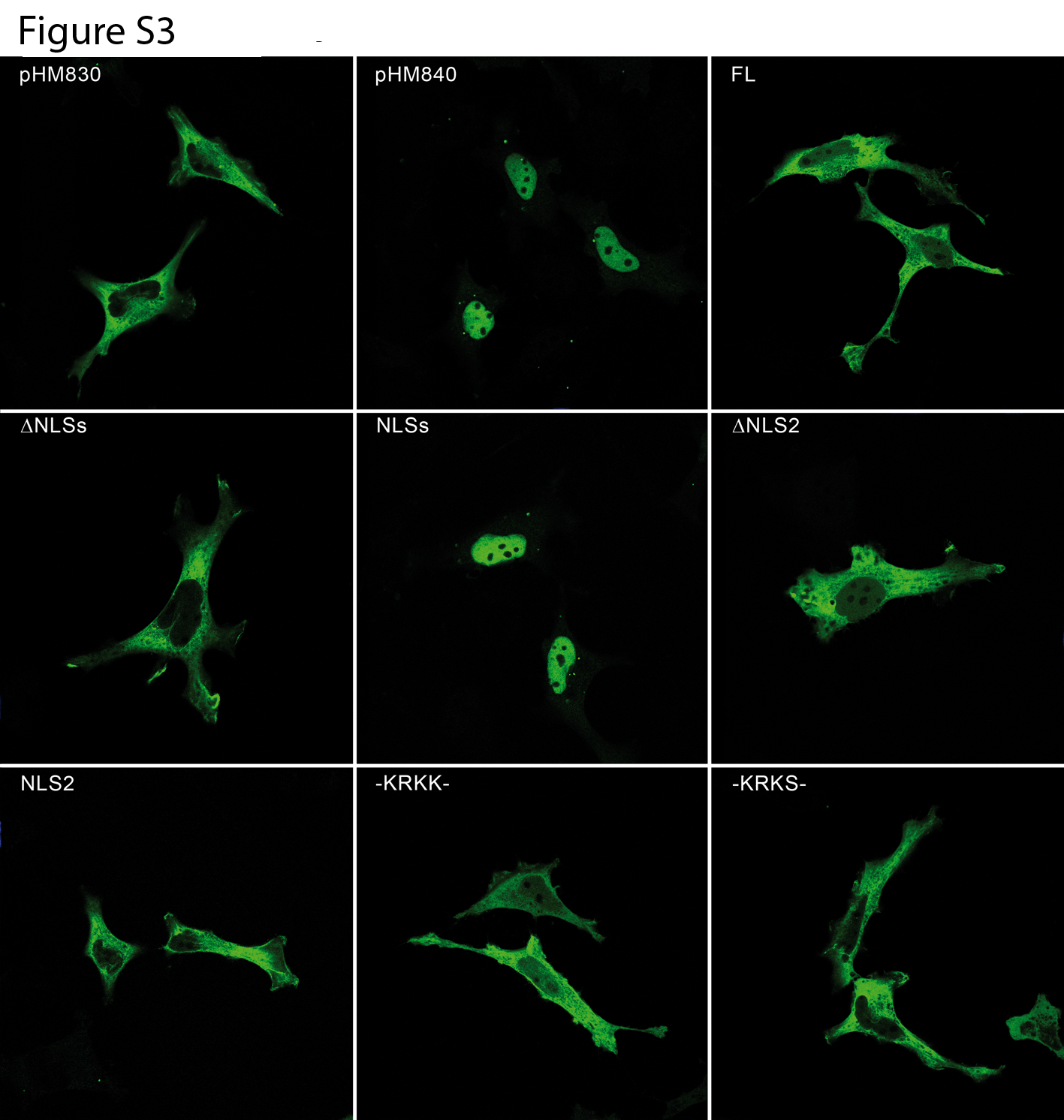

Supplement: Figure S3 — Confocal images of transfected HeLa cells expressing GFP-β-Gal NLS constructs and their controls. Distribution of GFP fusion proteins is shown. KRKK, represents a construct encompassing precise residues from NLS1 of BRMS1 protein resembling the location pattern of the full length (FL). KRKS is a point mutant where the last positively charged residue shifted to uncharged, which was unable to relocate the heterologous protein to the cell nucleus. (6.37 MB TIF) [file pone.0006433.s003.tif]

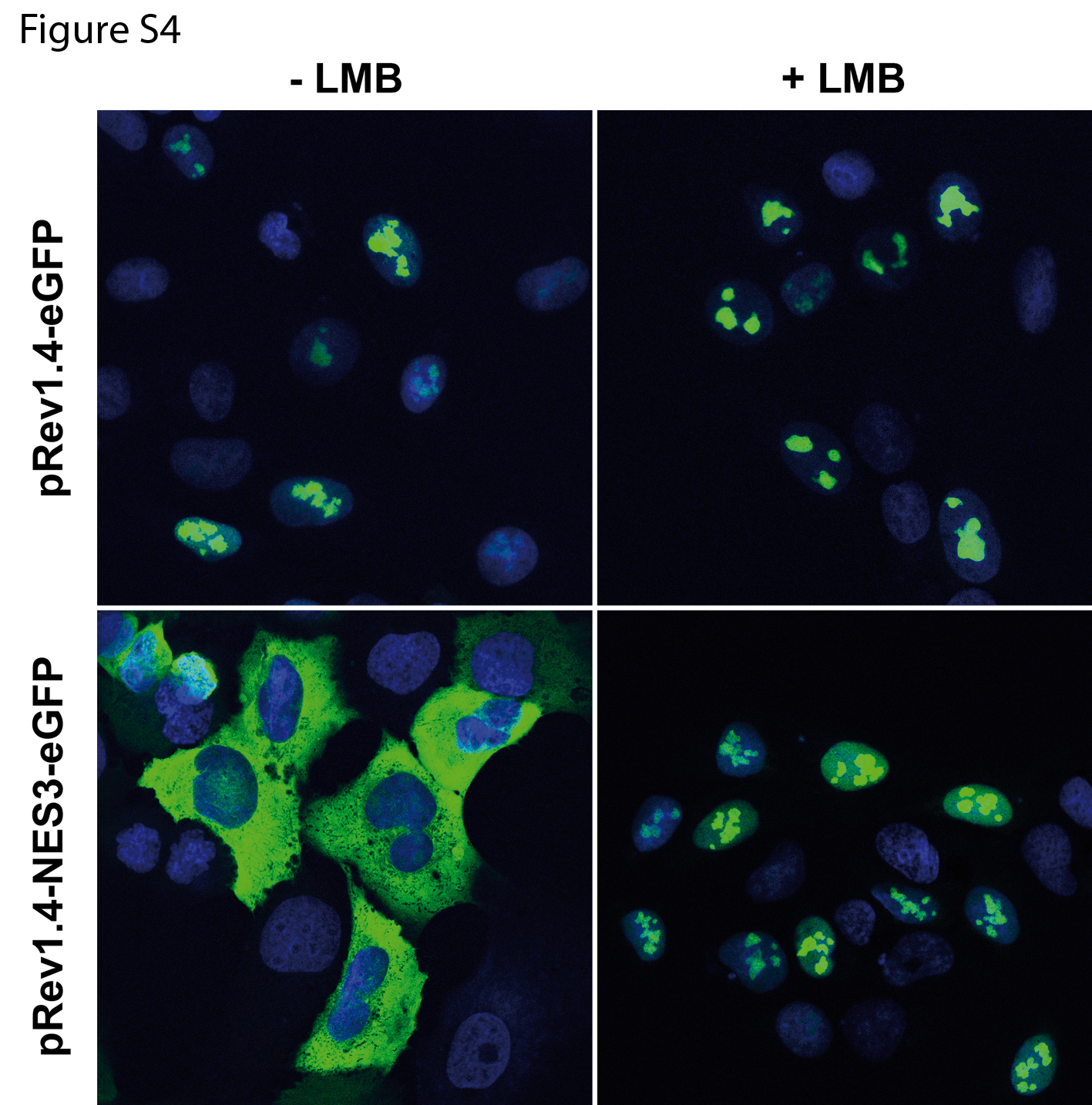

Supplement: Figure S4 — The pRev1.4-eGFP and pRev1.4-NES3-eGFP fusion proteins were transiently over-expressed in U-2 OS cells. Cell samples were untreated (-) or treated (+) with LMB to assess CRM1 transporter inhibition. (6.13 MB TIF) [file pone.0006433.s004.tif]
